# Cognitive Function, Sleep, and Neuroinflammatory Markers in Mice Exposed to Very Long-Term Intermittent Hypoxia

**DOI:** 10.3390/ijms26051815

**Published:** 2025-02-20

**Authors:** Clementine Puech, Mohammad Badran, Max B. Barrow, David Gozal

**Affiliations:** 1 Department of Child Health, Child Health Research Institute, School of Medicine, University of Missouri, Columbia, MO 65201, USA; clementinepuech@oulook.com (C.P.); mbadran@health.missouri.edu (M.B.); mbbhww@umsl.edu (M.B.B.); 2 Department of Pediatrics and Office of the Dean, Joan C. Edwards School of Medicine, Marshall University, Huntington, WV 25701, USA

**Keywords:** aging, obstructive sleep apnea, intermittent hypoxia, cognitive function

## Abstract

Chronic intermittent hypoxia (IH) is one of the hallmark features of obstructive sleep apnea (OSA) and adversely affects neurocognitive and behavioral functioning. However, how the duration of IH correlates with its deleterious effects remains unexplored. We aimed to assess the effects of IH over a prolonged period of time mimicking untreated OSA. Male C57Bl/6J mice were exposed to IH for 96 weeks. Sleep activity was acquired using a piezoelectric system. Novel object recognition (NOR) and the elevated plus maze test (EPMT) were conducted as measures of cognitive function and anxiety, respectively. Brain inflammation was evaluated by a panel of inflammation marker assays. All tests were performed after 16 and 96 weeks of IH exposure. After 96 weeks, sleep percentages during the dark phase decreased in both IH and room air (RA) compared to 16-week exposure (RA: *p* = 0.0214; IH: *p* = 0.0188). In addition to age-dependent declines in NOR performance, the mice after 96 weeks of IH exposure had lower NOR preference scores than RA controls (*p* = 0.0070). The time spent in open arms of the EPMT was reduced in mice exposed to IH compared to RA. Inflammatory marker expression increased in IH-exposed mice. Thus, aging and IH induce similar alterations in sleep, cognition, and neuroinflammation. However, the effects of aging are exacerbated by concurrent IH, suggesting that OSA is a disease associated with an acceleration in biological aging.

## 1. Introduction 

Obstructive sleep apnea (OSA) affects nearly one billion people worldwide and constitutes a major health concern [1,2,3]. OSA is characterized by the repetitive occurrence of either partial or complete upper airway obstruction during sleep. Recurrent collapse of the upper airway develops episodically with sleep onset and is associated with both intermittent hypoxia (IH) and recurring arousal leading to sleep fragmentation (SF) [4,5,6]. OSA is associated with an enhanced risk for the presence of excessive daytime sleepiness (EDS), which can affect daytime functioning including reducing attention span, cognitive dysfunction, fatigue, irritability, and depressive symptoms. All these and other consequences of OSA lead to poor health outcomes, jeopardize quality of life, and ultimately result in increased overall mortality [7]. Despite such ominous consequences, it is estimated that more than 80% of OSA patients remain undiagnosed, and therefore untreated [8]. 

Some patients can spend months or years with the disease without being aware of its presence because symptoms can be easily overlooked or be attributed to something else less serious. Some of the consequences of OSA can easily be misconstrued for aspects of aging or manifest in the context of chronic diseases such as depression, heart disease, diabetes, or dementia [9,10,11]. Additionally, OSA prevalence increases with aging, and older adults may not necessarily know about it or report symptoms such as loud snoring or witnessed apneas because they live alone or assume these symptoms are not worth reporting. One of the most notable changes with aging is the reduction in slow-wave sleep [11,12,13], and, consequently, older adults often experience less restorative sleep and feel unrefreshed after sleep, contributing to daytime fatigue and reduced cognitive performance [14,15]. Changes in sleep patterns can slow cognitive processing, making it harder to focus on tasks, process information quickly, and make decisions. Conversely, cognitive decline can further disrupt sleep, creating a cycle that impacts overall health and quality of life [16]. Aging is accompanied by chronic low-grade inflammation [16,17,18,19], and in the brain, aging promotes microglial activation along with excessive production of reactive oxygen species (ROS) resulting in oxidative stress [16,20,21] and neuroinflammation [22,23]. Thus, aging is accompanied by the release of pro-inflammatory cytokines and activation of key inflammatory pathways, such as the nuclear factor-kappa B (NF-κB) pathway [21,24]. Chronic neuroinflammation impairs synaptic plasticity [25,26,27], ultimately affecting cognitive functions, including memory, attention, and executive function, leading to cognitive decline.

As mentioned, the risk of developing OSA rises with age [28,29]. The ability of the central nervous system (CNS) to cope with stress and repair itself declines with age, likely exacerbating the adverse impact of IH [30]. Untreated OSA can exacerbate many aging-related health issues, and the interplay between undiagnosed OSA and aging is complex, not well understood, and organ-specific [13,31]. Furthermore, the cognitive changes imposed by concurrent aging associated with undiagnosed sleep apnea have not been thoroughly explored. The cumulative effects of poor sleep, cognitive decline, cardiovascular issues, and mood disturbances can lead to a loss of independence. In this setting, murine models of OSA have clearly demonstrated that OSA can impair memory, sleep, and brain inflammation [6,21,32]. Our objectives were to study the effects of untreated long-term IH associated with aging by assessing potential associations between neuroinflammatory markers, sleep, and cognitive function in mice chronically exposed to lifetime IH patterns mimicking moderate to severe OSA compared to room air (RA) littermates. To the best of our knowledge, the current study is the first to examine this issue and provides compelling evidence that even though the deterioration of cognitive functions slows down with extended exposures, the effect is still present and measurable if sufficient exposure duration is allowed.

## 2. Results

**Figure 1:**
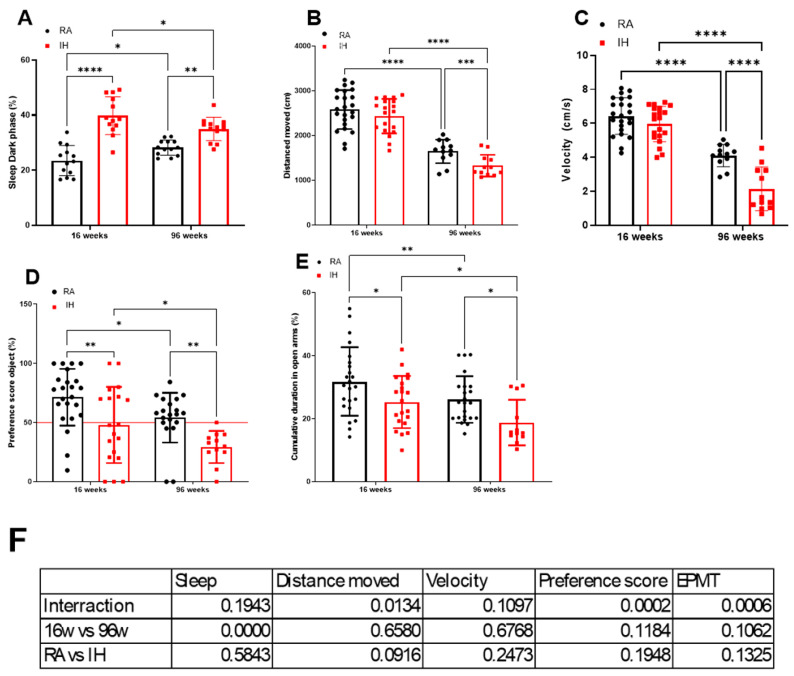
Sleep and cognitive function in mice exposed to IH for 16 weeks and 96 weeks. Sleep patterns during the dark phase n = 12–13/group (**A**), distance moved n = 12–23/group (**B**), and velocity n = 12–23/group (**C**) during the open field test, preference score during the NOR test n = 12–23/group (**D**), and cumulative duration in the open arms during the EPMT n = 12–23/group (**E**). Data are shown as the mean ± SD, n = 12–20/group. * *p* < 0.05, ** *p* < 0.01, *** *p* < 0.001, **** *p* < 0.0001. (**F**) Effect size for each measure of the η^2^ in each of the experiments.

Mice were exposed to IH for 96 weeks, and sleep and cognitive functions were evaluated at 16 weeks of exposure (IH16W) and then again at 96 weeks (IH96W) to examine whether any salient differences would emerge between mid-term and long-term IH as far as its effects on sleep, spontaneous mobility and velocity, explicit memory, and anxiety (Figure 1).

Sleep during the dark phase was increased in IH-exposed mice at both 16 weeks (39.8 ± 6.9%) and 96 weeks (35.0 ± 4.2%) when compared to their time controls (RA16W: 23.5 ± 5.5%, *p* < 0.0001; RA96W: 28.2 ± 2.8%, *p* = 0.0014). Of note, the percentage time spent in sleep during the dark phase increased in RA mice with age (RA16W vs. RA96W: *p* = 0.0214) while it decreased in IH-exposed mice (IH16W vs. IH96W: *p* = 0.0188) (Figure 1A).

With aging, both IH- and RA-exposed mice showed significant decreases in the distance covered during the open field tests (RA16W: 2583 ± 1 cm vs. RA96W: 1648.4 ± 264.8 cm, *p* < 0.0001; IH16W: 2435 ± 386.7 cm vs. IH96W: 1329.0 ± 242.9 cm, *p* < 0.0001). However, only mice exposed for 96 weeks exhibited significant decreases in mobility when compared to their time controls (RA96W vs. IH96W: *p* = 0.0007) (Figure 1B). With aging, the velocity during the open field experiment was also reduced (RA16W: 6.4 ± 1 cm/s vs. RA96W: 4.1 ± 0.7 cm/s, *p* < 0.0001; IH16W: 5.9 ± 1 cm/s vs. IH96W: 2.1 ± 1.7 cm/s, *p* < 0.0001), with no differences in velocity at the 16-week exposures but significantly lower velocities recorded in the IH96W mice (RA96W vs. IH96W: *p* < 0.0001) (Figure 1C).

In the novel object recognition (NOR) tests, the preference scores were significantly lower after both 16 weeks (48.8 ± 3%) and 96 weeks (29.43 ± 13.6) of IH exposure compared to their time-control counterparts (RA16W: 71.4 ± 24.0%, *p* = 0.0029; RA96W: 54.2 ± 21.1%, *p* = 0.0070). In both the RA and IH mice, aging was associated with reduced scores (RA16W vs. RA96W: *p* = 0.024; IH16W vs. IH96W: *p* = 0.0420). Of note, a 24% in the preference score occurred in RA-exposed mice with aging, while in IH-exposed mice the reduction in NOR performance was ~39% (Figure 1D).

In the elevated plus maze test (EPMT), the time spent in open arms in mice exposed to IH decreased after 16 weeks (25.31 ± 8.3%) and 96 weeks (18.8 ± 7.2%) compared to corresponding controls (RA16W: 31.8 ± 10.9%, *p* = 0.0167; RA96W: 26.1 ± 7.4%, *p* = 0.0181). With aging, both RA and IH mice spent significantly less time in the open arm (RA16W vs. RA96W: *p* = 0.0043; IH16W vs. IH96W: *p* = 0.0114) (Figure 1E).

**Figure 2:**
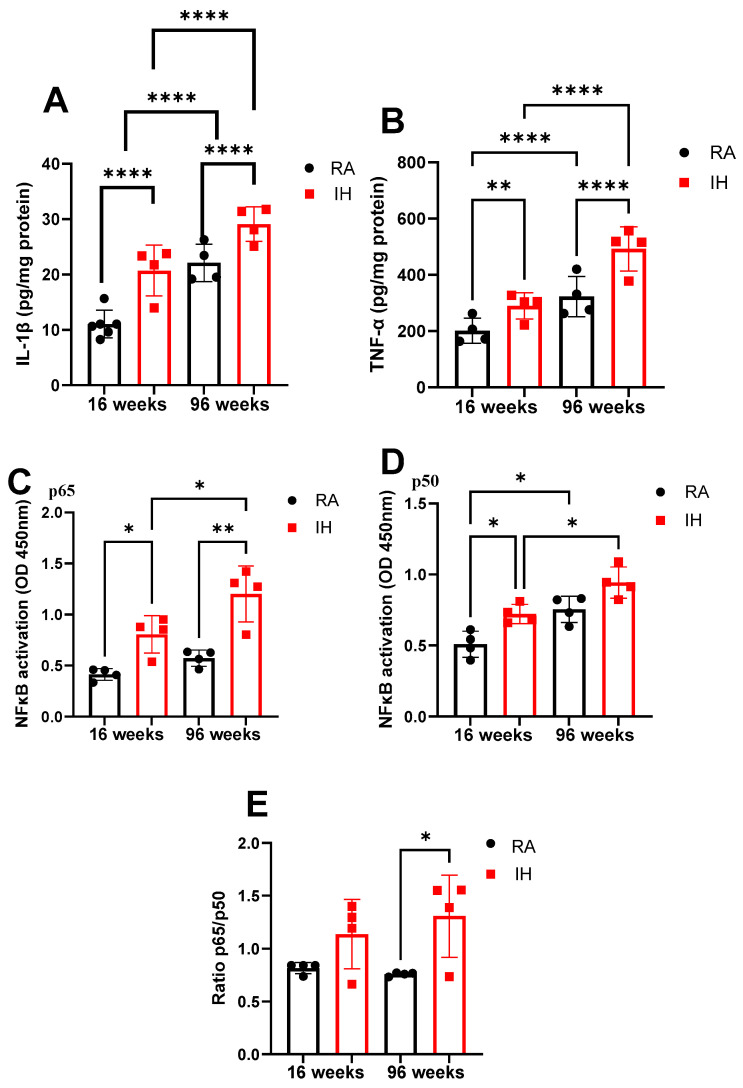
Inflammatory markers in the brain after 16 or 96 weeks of intermittent hypoxia during daylight hours. IL-1 β (n = 4–6/group) (**A**), TNF-α (n = 4/group) (**B**), NF-κB p65 subunit (n = 4/group) (**C**), NF-κB p50 subunit (**D**), and NF-κB ratio p65/p50 (n = 4) (**E**). Data are shown as the mean of triplicates for each mouse ± SD. * *p* < 0.05, ** *p* < 0.01, **** *p* < 0.0001.

Interleukin 1β levels were increased in IH16W mice (20.7 ± 4.6 pg/mL) when compared to RA16W mice (11.2 ± 3.2 pg/mL; *p* < 0.0001) with further increases with aging as illustrated by concentrations measured after 96 weeks (RA96W: 22.1 ± 3.4 pg/mL; IH96W: 29.1 ± 3.1 pg/mL; *p* < 0.0001). The effect of aging was similar across RA and IH conditions (Figure 2A).

TNF-α levels were higher at both 16 and 96 weeks of IH- exposures (IH16W: 322.7 ± 71.6 pg/mL; IH96W: 492.8 ± 78.6 pg/mL) compared to their time-control counterparts (RA16W: 201.7 ± 44.74 pg/mL, *p* = 0.0041; RA96W: 289.9 ± 46.3 pg/mL, *p* < 0.0001). Aging led to increased TNF-α levels (*p* < 0.0001), and the aging effect was similar across both RA and IH conditions (Figure 2B).

Transcriptional activity of the NF-κB p65 subunit increased with IH exposure (IH16W: 0.6 ± 0.08 A.I. vs. RA16W: 0.4 ± 0.05 A.I., *p* = 0.0329; IH96W: 1.2 ± 0.3 A.I. vs. RA96W: 0.8 ± 0.2 A.I., *p* = 0.0011). Aging was accompanied by an increased NF-κB p65 subunit in mice exposed to IH (*p* = 0.0303). The NF-κB p50 subunit increased significantly only in the IH-mice exposed for 16 weeks (0.7 ± 0.09 A.I.) compared to RA (RA16W: 0.5 ± 0.09 A.I., *p* = 0.0292) (Figure 2C). With aging, both RA- and IH-exposed mice showed increases in the NF-κB subunit p50 level (RA96W: 0.7 ± 0.06 A.I. vs. RA16W: *p* = 0.012; IH96W: 0.9 ± 0.11 vs. IH16W, *p* = 0.0227) (Figure 2D).

The ratio of p65/p50 NFκB sub-units showed no significant differences between IH and RA after 16 weeks of exposure or with aging. After 96 weeks of IH exposure, the p65/p50 NFκB ratio increased (1.3 ± 0.4) when compared to RA96W (0.8 ± 0.02, *p* = 0.0441) (Figure 2E).

**Figure 3:**
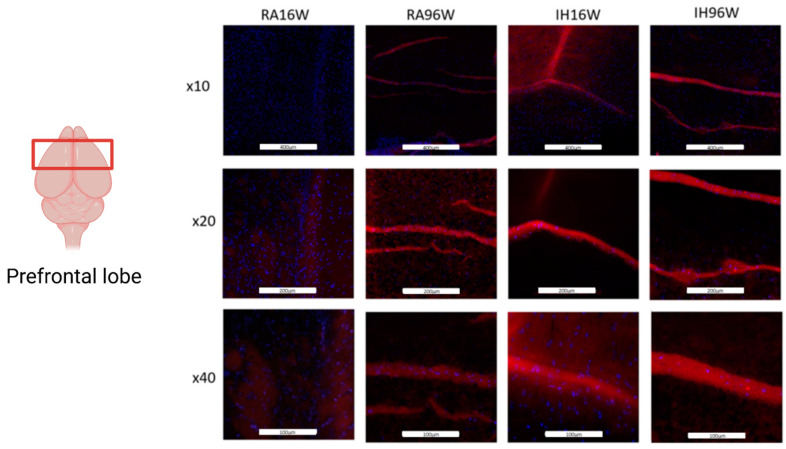
Immunostaining for p16 (red) as a senescence marker in prefrontal lobe brain sections after 16 or 96 weeks of IH or RA exposures. The nuclei were counterstained by DAPI (blue). n = 4/condition. White bars indicate 400 micron for ×10 magnification, 200 micron for ×20 magnification and 100 micron for ×40 magnification.

The immunostaining of IH-exposed mice brain cortex revealed increases in P16 immunoreactivity. Of note, the p16 immunoreactivity after 16 weeks of IH was remarkably similar to that of mice exposed to room air for 96 weeks (Figure 3). 

**Figure 4:**
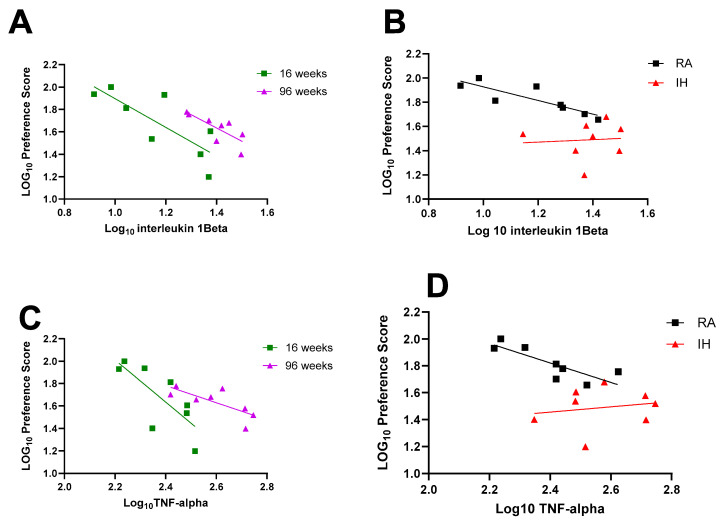
Correlations between NOR test preference score and interleukin 1β (**A**,**B**) at 16 weeks (n = 8, r Spearman = −0.7381, Y = −1.281 + 3.177, *p* = 0.0458, R^2^ = 0.6343) in green and at 96 weeks (n = 8, r Spearman = −0.7857, Y = −1.184x + 3.292, *p* = 0.0279, R^2^ = 0.6002) in purple (panel (**A**)) in RA-exposed mice (n = 8, r Spearman = −0.9524; Y = −0.5634x + 2.490, *p* = 0.0011, R^2^ = 0.735) in black and in IH-exposed mice (n = 8, r Spearman = 0.2381, Y = 0.1009x + 1.349, *p* = 0.5821, R^2^ = 0.0057) in red (panel (**B**)). Correlation between NOR test preference scores and TNF-α (**C**,**D**) at 16 weeks (n = 8, r Spearman = −0.7619, Y = −1.881x + 6.149, *p* = 0.0368, R^2^ = 0.5686) in green and at 96 weeks (n = 8, r Spearman = −0.7619, Y = −0.7611x + 3.608, *p* = 0.0368, R^2^ = 0.5657) in purple (panel (**C**)) in RA-exposed mice (n = 8, r Spearman = −0.8095, Y = −0.7248x + 3.560, *p* = 0.0218, R^2^ = 0.6753) in black and in IH-exposed mice (n = 8, r Spearman= −0.04765, Y = 0.1959x + 0.9850, *p* = 0.9349, R^2^ = 0.0331) in red panel (**D**).

To evaluate any potential association between neuroinflammation and cognitive functioning, IL-1β values for each mouse were plotted against corresponding NOR performance and revealed robust inverse correlation at 16 weeks (r Spearman = −0.7381, Y = −1.281 + 3.177, *p* = 0.0458) and at 96 weeks (r Spearman = −0.7857, Y = −1.184x + 3.292, *p* = 0.0279) (Figure 4A). In RA-exposed mice, IL1β levels were increased with aging, with corresponding declines in NOR performance (r Spearman = −0.9524; n = 8; Y = −0.5634x + 2.490, *p* = 0.0011). 

Linear regression between TNF-α levels and corresponding NOR preference scores corroborated the inverse correlation between neuroinflammation and cognitive function at 16 weeks (r Spearman = −0.7619, Y = −1.881x + 6.149, *p* = 0.0368) and 96 weeks (r Spearman = −0.7619, Y = −0.7611x + 3.608, *p* = 0.0368) indicating that as TNF-α levels increase, the more likely that NOR performances will be reduced (Figure 4C). With aging in RA mice, there is a strong negative correlation between TNF-α levels and NOR scores (r Spearman = −0.95, Y = −0.7248x + 3.560, *p* = 0.0218), which is lost in IH-exposed mice (Figure 4D).

## 3. Discussion

In the present study, we extended IH exposures to explore the impact of untreated OSA-like disease during a substantial portion of the lifespan. We found that IH induces inflammatory processes in the brain accompanied by substantial deficits in explicit memory function, as illustrated by the NOR test. We further identified that IH promotes increased expression of p16, a senescence marker, and that the effect of IH appears to be equivalent to 80 weeks of normoxic life (96 weeks in RA minus 16 weeks in IH), further corroborating the previous observations that untreated OSA is associated with an increase in biological aging markers [28,33]. p16 (also known as p16INK4a) is the gene encoding for the p16 protein that has emerged as a reliable senescence marker. P16 is involved in regulating the cell cycle, and its expression increases as cells undergo senescence, a state of permanent cell cycle arrest. As cells experience stress, such as DNA damage or telomere shortening, p16 expression increases, serving as a hallmark of senescence. Elevated p16 expression levels are associated with aging, tissue dysfunction, and the development of age-related diseases. Furthermore, removing p16-positive senescent cells delays the occurrence and progression of age-related pathologies in mice [33,34,35].

In addition, evidence for aging-induced and IH-induced neuroinflammation, as attested by sustained increases in the putative transcriptional activity of NF-κB and the brain expression of IL-1β and TNF-α, suggests a robust association between phenotypic indicators of senescence (i.e., increased sleep propensity during the active circadian phase, reduced mobility and velocity, and diminished cognitive functioning) and biomarkers of neuroinflammation. Taken together, these results provide further corroborative evidence of the roles of chronological aging and diseases characterized by IH in eliciting chronic low-grade inflammatory responses that ultimately result in diminished function and accelerated biological aging. 

The relationship between aging, sleep, and cognition is complex, with each of these influencing the other two in significant ways. As we age, changes in sleep patterns can contribute to cognitive decline, while cognitive issues can further disrupt sleep [9,35,36]. According to the previous literature, patients with OSA show significant impairments in vigilance, executive function, and memory [37,38,39]. Since OSA prevalence increases with chronological age, these cognitive impairments are often observed in older people, thereby raising the possibility that aging rather than OSA underlies these functional deficits. However, OSA leads to impairments in performance and brain function in young people including children, thereby negating the assumption that the preponderant contribution to such deficits exclusively originates in the aging process [40,41]. Here we show that long-term IH can accelerate cognitive aging, leading to earlier onset of age-related cognitive declines and worse NOR scores when compared to chronologically age-matched mice [42]. The findings in these animal models are congruent with recent findings associating untreated OSA with aging-related cognitive deficit [43], as well as with increased vulnerability to early stages of dementia and the development of neurodegenerative diseases [37]. Previous studies from our group in mice using similar IH exposure profiles identified stabilization of the performance in NOR tests from 45 days to 240 days of exposure [44]. However, cessation of exposure at this particular time versus the current prolongation of exposure to 672 days, as conducted in the present study, does not permit adequate comparisons to be made on the interactions between chronological aging and IH-mediated effects on explicit memory performance measures. 

The relationships between untreated OSA and brain inflammation are complex and driven primarily by the effects of IH and sleep disruption. Persistent neuroinflammation may impair synaptic plasticity and lead to cognitive decline, manifesting as difficulties with attention, memory, executive function, and mood disorders including anxiety and depression [26,27]. These alterations are remarkably similar to those identified during chronological aging. Moreover, chronic brain inflammation associated with sleep disorders has been linked to the accumulation of neurotoxic proteins in the brain and a high risk of developing dementia and Alzheimer’s disease (AD) [45,46]. As illustrated by current experiments, the sleep disturbances induced by IH—and IH itself—are accompanied by chronic inflammation that likely contributes to mood disorder and cognitive deficits, thereby creating a vicious cycle. NF-κB activity and the corollary increases in the production of proinflammatory cytokines such as TNF-α and IL-1β illustrate the inflammatory responses associated with both chronological aging and IH as the driver of accelerated biological aging. We should point out that activation of the NF-κB signaling pathway can also be induced by stressful stimuli, and it is likely that IH operates as a chronic stressor in our murine model of OSA [47]. It is likely that prolonged neuroinflammation might eventually lead to the release of excessive inflammatory cytokines and chemokines and may cause neuronal cell death and result in cognitive impairments [48,49]. In a previous study, we showed that disrupted sleep may induce increased levels of pro-inflammatory cytokines such as TNF-α in mice that closely correlate with sleep propensity during the active circadian phase, similar to current findings using IH as the exposure [50]. Furthermore, analogous findings have been reported in adults and children suffering from OSA [51,52,53]. Similar circumstances appear to be operational in aging, whereby elevation of inflammatory markers is associated with neurodegenerative changes [16]. Age-related neurodegenerative processes are attributable to increased neuronal vulnerability and protein aggregation that are accelerated by the alterations in the concentrations of cytokines and their cumulative impact on cognitive function [54,55]. Cognitive impairments are increasingly being linked to disruptions in sleep and chronic inflammation. Sleep plays a crucial role in memory consolidation, attention, and overall brain function, and poor sleep can impair learning and lifestyle [13,56]. Sleep is a fundamental physiological function involved in the restoration of cellular and organ homeostasis and is essential for optimal daytime functioning. Disrupted sleep has also been associated with a higher risk of neurodegenerative diseases like Alzheimer’s and Parkinson’s. The relationship between sleep disruption and inflammation is complex, with poor sleep leading to increased inflammation, and inflammation, in turn, negatively affecting sleep quality, creating a vicious cycle that can worsen cognitive decline. Accumulating evidence has linked aging and sleep to cognitive decline and risk of dementia [57,58,59]. Normally, inflammation is a protective response that facilitates the healing process; however, prolonged inflammation can cause tissue damage. Normal aging is associated with heightened prolonged inflammation throughout the brain that alters cognitive function. Persistent increased levels of inflammation are associated with neurodegeneration disease.

Before we conclude, it is important to denote several limitations of the current study. Due to the large number of mice required, it was not feasible to extend our experiments to include additional exposure durations and recovery periods before completing the current observations. This is why we did not explore different time points between 16 and 96 weeks. It will be interesting to assess if, between 16 weeks and 96 weeks, there is an adaptive effect of the rodents to the intermittent hypoxic environment for at least a period of time [60]. Additionally, we only used male mice, such that the effects of prolonged intermittent hypoxia (IH) exposures on females remain unexplored. Since the animals were young at the start of the exposure, it would also be valuable to study older animals over a long period. Finally, we did not investigate the potential mechanisms underlying the harmful effects of IH on the blood–brain barrier (BBB) and cognition. Furthermore, we did not investigate different brain regions that exhibit differential susceptibility. Our focus was solely on the effects of IH exposure, and further research will be needed to assess the impact of other aspects of obstructive sleep apnea (OSA), such as sleep fragmentation, episodic hypercapnia, and increased intrathoracic pressure fluctuations, either individually or in combination. Therefore, a future study could focus on investigating the long-term effects of intermittent hypoxia (IH) exposure on the blood–brain barrier (BBB) and cognitive functions in both male and female mice. The study should also aim to uncover the underlying mechanisms driving the harmful effects of IH on the BBB and cognition, particularly focusing on molecular pathways such as inflammation, oxidative stress, and endothelial dysfunction. The hippocampus and its role in the inflammation and learning processes could also be investigated [61].

In summary, given the profound impact that undiagnosed OSA can have on biological aging and its consequences, early detection and treatment are crucial. Undiagnosed OSA in older adults is a serious health concern that can accelerate the aging process and exacerbate age-related health issues. The combination of age-related vulnerabilities and the inflammatory effects of untreated OSA can accelerate cognitive decline, worsen underlying neurodegenerative diseases, and impact mental health, while, in parallel, adversely promoting other system-related morbidities and enhancing overall mortality [62,63].

## 4. Materials and Methods

### 4.1. Animal Model

All the experiments were approved by the Institutional Animal Care and Use Committee (IACUC) of the University of Missouri (protocol # 38864). Fifty male C57BL/6 J mice (8 weeks old) were purchased from Jackson Laboratory (Bar Harbor, ME, USA). The animals were housed in a controlled environment with 12 h light–dark cycles (6 a.m. to 6 p.m.) at a constant temperature (24 ± 0.2 °C) with *ad libitum* access to food (normal chow) and water. All the animals were allowed to fully acclimate within the animal care facility for seven days upon arrival. The animals were then randomized to normoxic (RA) or IH exposure. The exposure lasted for a total of 96 weeks. 

#### Intermittent Hypoxia (IH)

The IH exposure protocol used has been previously described in detail [64,65,66]. The mice were exposed to IH for 96 weeks, and sleep and cognitive functions were evaluated at 16 weeks of exposure (IH16W) and then again at 96 weeks (IH96W) (for those remaining after euthanasia of a proportion of the mice for tissue assessments). In brief, the mice were subjected to IH, while room air (RA)-exposed control mice were housed in standard housing conditions and exposed to normoxic gas. IH exposure consisted of alternating 21% FIO_2_ and 6.5% FIO_2_, 20 cycles h−1 for 12 h day−1 during daylight (06:00 h–18:00 h) using a commercially available system (80 × 50 × 50 cm; Oxycycler A44XO, BioSpherix, Redfield, NY, USA). The IH exposure revealed nadir oxyhemoglobin saturations in the range of 68–75%, which are the primary correlate of moderate to severe OSA in humans [67]. All the mice were maintained in normoxic conditions (21% FIO_2_) during the dark period (from 18.00 h–06.00 h). The RA mice were maintained in normoxic conditions (21% FIO_2_) throughout the exposure duration.

### 4.2. Behavioral Tests

The behavioral experiments were performed by operators who were blinded to the exposure and were conducted by three observers between 9 a.m. and 5 p.m. The behavioral test battery consisted of the novel object recognition (NOR) test, and the elevated plus maze test (EPMT). Throughout the experiments, the experimental set-ups were cleaned with 70% ethanol to prevent odor cues. All the mazes were purchased from Maze Engineers (Cambridge, MA, USA). The tests were recorded from a vertical point of view with a video camera suspended above the experimental area. All the experiments were interfaced with a video tracking system (Noldus Ethovision XT17 Software, Leesburg, VA, USA).

#### 4.2.1. Open Field

The open field is a test used for measuring locomotor activity and behavior in laboratory rodents [66,68,69]. We used the habituation phase of the NOR test described above to measure the locomotor activity of the mice. The animals were allowed to explore the open field for 10 min, with locomotor activity monitored during the first 5 min. The distance moved and velocity were analyzed and quantified by the tracking software.

#### 4.2.2. Novel Object Recognition (NOR) Test

The NOR test has been previously extensively described [32,64,65,70]. Briefly, this experimental paradigm is used to evaluate explicit memory based on the innate tendency of mice to explore novelty settings. For each trial, mice were placed in the center of a blue opaque open-field plastic chamber. The NOR test is composed of three distinct phases. The habituation phase allows mice to explore the open field for 10 min. During the second phase of 5 min, two identical objects are placed in the arena. During the third and last phase, one of the objects is replaced by a new object. Different objects of different colors, shapes, and sizes were used as novel objects and placed randomly either on the left or on the right side of the arena. The mice were allowed to freely explore the objects for 5 min. Positive exploration by the mouse was defined as touching the object with the nose. The time spent exploring the objects was analyzed and quantified by the tracking software (Noldus Ethovision XT16 Software, Leesburg, VA, USA). The total exploration time for both objects was recorded. Results were reported as preference scores using the following formula [64,70,71]:

$$Preference\;score=\frac{Time\;spent\;near\;to\;novel\;object}{Time\;spent\;near\;to\;all\;objects}\times 100$$

The mice who did not explore objects were removed from the experiment. The animals were considered to have an explicit preference for novelty if their preference score was >50%.

#### 4.2.3. Elevated Plus Maze Test (EPMT)

The EPMT was used to assess anxiety behaviors [64,72,73,74]. The apparatus consists of an elevated cross formed by two open arms and two closed arms (arm length 35 cm, wall height for closed arms 20 cm) made of blue Plexiglas radiating from a central platform to form a plus sign. A 0.5 cm height wall was added to the open arms to prevent the mice from falling, especially when they change their motion direction. The device is situated 56 cm above the floor. In our experiments, we considered the open arms as well as the center zone. The open arms are considered by mice as a threatening area. The animals were placed in the central area facing one open arm and allowed to explore the maze for 5 min. The time spent in the open arms is commonly used as a measure of impulsivity, while the time spent in the closed arms is deemed to reflect anxiety-like behaviors in mice.

#### 4.2.4. Sleep Recording

Sleep activity was monitored using a previously validated, computerized piezoelectric system (PiezoSleep; Signal Solutions, Lexington, KY, USA). This is a noninvasive system that automatically scores sleep and waking states in mice (SleepStat; Signal Solutions, Lexington, KY, USA) [32,64,65,74,75,76]. Briefly, a piezoelectric film placed under the floor of the cages can continuously detect pressure variations. For all the sleeping postures of the mouse, pressure variations from breathing are detected. Sleep states are characterized by quasi-periodic signals with low variations in amplitude, whereas wakefulness and rest states are characterized by irregular transient and high-amplitude pressure variations corresponding to body movements and weight shifting. Signal features sensitive to the differences between the sleep and wake states are extracted from short-time pressure signal segments, and classification is automatically performed every 2 s. 

The system records pressure changes using a piezoelectric film, the duration and intensity of which are automatically scored by computer algorithms and classified as wake or sleep states. The Piezo system exhibits 90% accuracy compared to EEG/EMG-based sleep acquisition and scoring approaches and has been previously validated [76].

### 4.3. Inflammatory Factors 

After the completion of the IH or RA exposure, the mice were euthanized by the introduction of 100% carbon dioxide into a euthanasia chamber. Then, their brains were immediately dissected, flash-frozen in liquid nitrogen, and stored at −80 °C until assayed. The transcriptional activity of NF-κB (Active motif kit; Carlsbad, CA, USA) and TNF-α levels (Sinobiological Labs; Beijing, China) was evaluated in the prefrontal cortical sections with commercially available kits, according to manufacturer instructions. IL-1β expression levels were assayed with a commercial kit from Abcam (Cambridge, UK). Every sample was normalized by protein quantification. The quantification was measured with purified protein in each kit. 

### 4.4. Immunohistochemistry

The mice were euthanized and transcardially perfused with PBS containing heparin-20U to wash out residual blood from cerebral circulation [32]. The mice were further perfused with 4% PFA in PBS and post-fixed in 4% PFA overnight at 4 °C. To prepare frozen sections, the post-fixed brains were immersed for 2–3 days in 20% (*w*/*v*) sucrose in PBS for cryoprotection. Then, the brains were sectioned in two, and each portion was placed in a cryomold and embedded in OCT (Tissue-Tek optimal cutting Temperature, Thermo Fischer, Hanover Park, IL, USA) frozen in an isopentane dry ice bath. The brains were divided into 7 µm thickness sections on a Leica CM1850 cryostat, mounted on superfrost glass slides, and frozen at −80 °C until assayed. The slides were dried at room temperature for 30 min, then placed on an epitope retrieval solution immersed in boiling water for 3 min, cooled for 5 min, and then subjected to immunostaining. First, the non-specific binding sites were blocked for 1 h at 4 °C in a blocking solution comprising 1% goat serum, 10% donkey serum, and 1% BSA in PBS. Then, the primary antibody (Rb pAb to CDKN2A/P16Ink4a-AB189034 Abcam) was diluted at 1/100 in 1% BSA in PBS and incubated overnight at 4 °C in a humid atmosphere. The slides were then washed three times for 5 min in PBS at room temperature. The secondary antibody (donkey anti-rabbit IgH Alexa Fluor 555-A32794 Invitrogen Rockford, IL, USA) was diluted at 1/1000 in 1% BSA PBS and incubated at room temperature in a humid atmosphere for 2 h and washed 3 times for 5 min in PBS. Then, the nuclei were counterstained with Dapi at 1/1000 for 10 min and washed 3 times for 5 min in PBS. The last wash was performed with water, and the slides were then covered with a mounting medium followed by the placement of a cover slide. The slides were then sealed with nail polish. The sections were then imaged using an epifluorescence microscope (Echo Confocal microscope; Echo, San Diego, CA, USA).

### 4.5. Statistical Analysis

Statistical analysis was performed using Prism 9.2 for Windows (GraphPad Software, San Diego, CA, USA www.graphpad.com). Two-way ANOVA and mixed models followed by Fisher post hoc tests were used, and the data were expressed as the mean ± SD. A *p*-value < 0.05 was considered statistically significant. η^2^ was used as representative of the effect size after the ANOVA and mixed models. Mixed models were used for the NOR test since this test does not follow the normality distribution. For the correlation test, the non-parametric Spearman test was performed. Because of the non-normal distribution of the preference score, R^2^ was used as the effect size.

## Data Availability

Data is available upon request from the corresponding author at his discretion since the data are still being analyzed in other related projects.
